# Clinical use of color Doppler ultrasonography to predict and evaluate the collateral development of two common revascularizations in patients with moyamoya disease

**DOI:** 10.3389/fneur.2022.976695

**Published:** 2022-10-28

**Authors:** Jing-Zhe Wang, Jie Mu, Dong Zhang, Shuai Zheng, Xun Zhu, Xi Wei

**Affiliations:** ^1^Diagnostic and Therapeutic Ultrasonography Department, Tianjin Medical University Cancer Institute and Hospital, National Clinical Research Center for Cancer, Key Laboratory of Cancer Prevention and Therapy, Tianjin, China; ^2^Tianjin's Clinical Research Center for Cancer, Tianjin, China; ^3^Ultrasound Department, Beijing Tiantan Hospital, Capital Medical University, Beijing, China; ^4^Neurosurgery Department, Beijing Tiantan Hospital, Capital Medical University, Beijing, China; ^5^Neurosurgery Department, The Second Hospital of Tianjin Medical University, Tianjin, China

**Keywords:** extracranial arteries, moyamoya disease, revascularization surgery, surgical collateral development, ultrasound

## Abstract

**Objective:**

To explore the value of color Doppler ultrasonography (CDU) to predict preoperatively and evaluate postoperatively the collateral development of two common revascularizations in patients with moyamoya disease (MMD).

**Methods:**

We prospectively enrolled 49 patients with MMD who underwent unilateral superficial temporal artery (STA) -middle cerebral artery (MCA) anastomosis or encephalo-duro-arterio-synangiosis (EDAS). The parameters of the extracranial arteries, including STA, internal carotid artery (ICA), external carotid artery (ECA), and vertebral artery (VA), were performed before and at 3–6 months after surgery. DSA results were used to assess surgical collateral development.

**Results:**

To predict good collateral development before STA-MCA anastomosis, the preoperative D > 1.75 mm in the STA had the highest area under the Receiver Operating Characteristic curve (AUC). To predict good collateral development before EDAS, the preoperative EDV > 12.00 cm/s in the STA had the highest AUC. To evaluate the good collateral development after STA-MCA anastomosis, the postoperative EDV > 16.50 cm/s in the STA had the highest AUC. To evaluate the good collateral development after EDAS, an increase of D of 0.15 mm in the STA had the highest AUC. Logistic regression analysis showed that the preoperative RI and EDV in the STA were highly correlated with collateral development. Besides, the preoperative RI was an independent risk factor for collateral development.

**Conclusion:**

CDU could predict preoperatively and evaluate postoperatively the collateral development of STA-MCA anastomosis and EDAS surgery postoperatively by detecting ultrasound parameters of the STA.

## Introduction

Moyamoya disease (MMD) is a rare cerebrovascular disease characterized by progressive steno-occlusion of the bilateral terminal internal carotid artery (TICA) or/and the origin of a middle cerebral artery (MCA) or/and anterior cerebral artery (ACA) (1, 2). The steno-occlusion leads to the reduction of cerebral perfusion, which could promote the formation of collateral circulation, such as the moyamoya vascular network at the base of the skull. When the collateral vessels are insufficient or ruptured, the symptoms of cerebral ischemia or hemorrhage will occur (3, 4). Currently, these symptoms can be relieved or treated by extracranial-intracranial bypass surgeries, including direct bypass, indirect revascularization, and combined surgeries. The two most common surgical procedures of these types are superficial temporal artery (STA) -MCA anastomosis and encephalo-duro-arterio-synangiosis (EDAS) (1, 5). Clinicians generally choose an appropriate surgical method according to the patient's hemodynamic data (6, 7).

Previous studies have shown that the increase in postoperative collaterals is closely related to the prognosis of patients (8, 9). However, the formation of collaterals is a process with great individual differences. More importantly, by supplying collaterals into the brain after surgery, the STA could also cause stenosis after a period of time (10, 11). Therefore, long-term, regular follow-up is still needed after surgery. Digital substraction angiography (DSA) is the gold standard for follow-up after revascularization surgery in MMD patients. The collaterals supplied by the STA after surgery are graded according to the criteria proposed by Matsushima et al. (12, 13). Other imaging modalities, such as computed tomography (CTA) and magnetic resonance imaging (MRA), could also reflect postoperative collateral vessels (14–17). However, these methods have inherent costs and/or risks for regular and close surveillance.

Color Doppler ultrasonography (CDU) could be used as a non-invasive imaging method to quantitatively monitor vascular parameters. A series of studies have also shown that the blood flow velocity and resistance index (RI) of STA monitored by CDU have significant changes in MMD patients after different kinds of revascularization surgeries, and the degree of change could indirectly reflect the postoperative collateral development (18–21). However, there is still a lack of targeted research on the collateral development of STA-MCA anastomosis and EDSA. Meanwhile, the ability of CDU to predict postoperative collateral development before surgery is still unclear.

In the study, 26 patients with unilateral STA-MCA anastomosis and 23 patients with unilateral EDAS were collected to observe the differences in the inner diameter (D) and hemodynamics in the ipsilateral external carotid artery (ECA), internal carotid artery (ICA), vertebral artery (VA), and STA before and after the surgery to explore the effect on the blood flow distribution in the anterior and posterior circulation of the brain. More importantly, we analyzed the clinical value of STA parameters on the preoperative prediction and postoperative evaluation for collateral development of STA-MCA anastomosis and EDAS by CDU.

## Methods

### Patients

**Figure 1:**
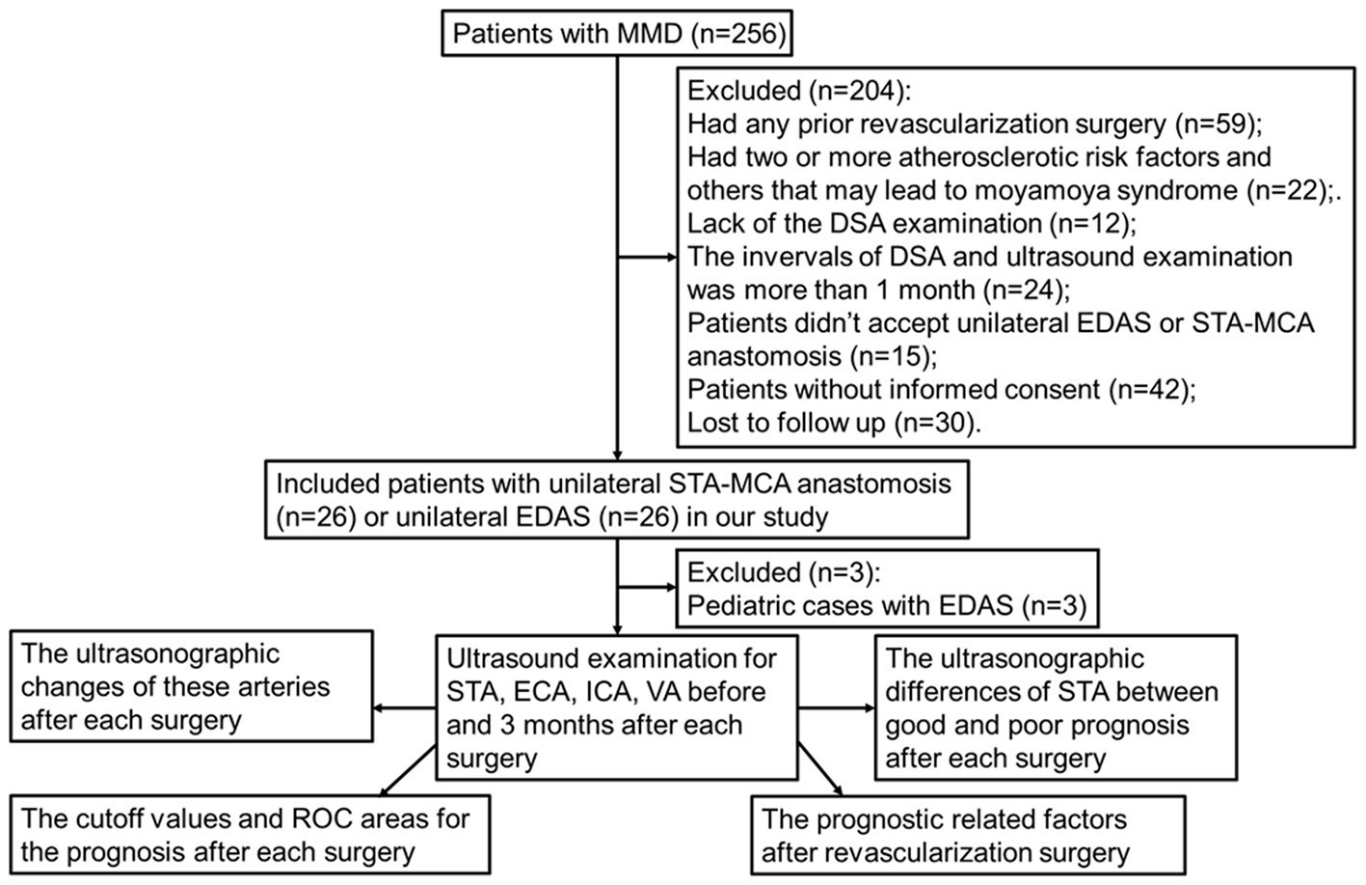
Schematic diagram of patient selection and exclusion. MMD, indicates moyamoya disease; DSA, digital subtraction angiography; EDAS, encephalo-duro-arterio-synangiosis; STA, superficial temporal artery; MCA, middle cerebral artery; ECA, external carotid artery; ICA, internal carotid artery; VA, vertebral artery.

Patients with MMD were enrolled in the neurosurgery department of the Beijing Tiantan Hospital, Capital Medical University, from April 2021 to March 2022. Both inpatients and outpatients were selectable for this study if they were diagnosed with bilateral MMD by DSA (22). We performed CDU as part of a routine workup for included patients within 1 month of the revascularization. The DSA and CDU examinations were performed 3–6 months after surgery, and the interval between the two examinations was < 1 month. Included patients who had no prior revascularization surgery. The patients were excluded from this study if the patients had two or more atherosclerotic risk factors and others that may lead to moyamoya syndrome. We excluded patients who lacked a CDU and/or DSA examination. Patients who did not accept STA-MCA anastomosis or EDAS were excluded from the study. Informed consent was required for all included patients. Finally, 23 patients with unilateral EDAS and 26 patients with unilateral STA-MCA anastomosis were enrolled in our study (Figure 1). The demographic data and clinical manifestations were recorded. The studies involving human participants were reviewed and approved by the Research Ethics Board of the Beijing Tiantan Hospital, Capital Medical University. Written informed consent to participate in this study was provided by the patients/participants.

### DSA

A DSA was performed and interpreted by a neuro-endovascular specialist. The 5F angio-catheter was placed at the C1 segment of the internal carotid artery (corresponding to the second cervical vertebra) according to the Seldinger method. The imaging parameters were four frames/s with injection (using a power injector, the pressure was 300 psi/kg) of 5 mL (3 mL/s) contrast medium for all series in all of the included patients in a single-plane angiographic machine (Artis zee floor, Siemens AG, Germany). According to the collateral development of the patients, patients who underwent cerebral revascularization were divided into the good collateral development group and the poor collateral development group. Good collateral development was defined as the lateral projection of the ECA angiogram showing that postoperative perfusion of STA collateral vessels exceeded 2/3 of the MCA blood supply area. In contrast, poor collateral development was defined as the lateral projection of the ECA angiogram showing that postoperative perfusion of STA collateral vessels was < 2/3 of the MCA blood supply area (12). DSA images of 49 patients were collected. Grading was determined by two radiologists blinded to the patient's clinical information and each other's interpretation.

### CDU

We performed the CDU examination using a color-coded ultrasound system (IU22, Philips Medical Systems, Bothell, WA, USA) with a 9–3 MHz linear-array transducer to examine STA and a 12–5 MHz linear-array transducer to examine ICA, ECA, and VA. The CDU parameters were measured 1 week before surgery and 3–6 months after surgery by an experienced ultrasound technician who was blind to clinical information, including DSA results. No contrast enhancement was used. During the ultrasound examination, the patient was placed in the supine position. The head was turned to one side to expose the neck. We measured the D and hemodynamic parameters of the ECA, ICA, VA, and STA on the side of revascularization surgery. The ICA was measured at 2.5–3.5 cm above the carotid bifurcation, and the ECA was assessed at 1.5–2.5 cm above the carotid bifurcation and the opening of the superior thyroid artery. The VA was analyzed at the level of the third intervertebral segment. The STA was assessed at the common STA segment at the level of the ear. In terms of the D of each vessel for the calculation of cross-sectional area, we obtained the D between inner luminal walls at the end-diastole. Probe pressure moderately during inspection to ensure data accuracy. All Doppler velocities were acquired at a Doppler angle of fewer than 60°. The hemodynamic parameters include end-diastolic velocity (EDV), time-averaged mean flow velocity (TAMV), RI, and flow volume (FV). The FV of each vessel was obtained from the TAMV of the cross-sectional area of the individual vessel; this formula has been built into the ultrasound system software. FV was calculated according to the following equation. FV = TAMV × π × D^2^/4 × 60.

### Statistical analyses

Continuous variables were presented as mean ± SD or median values (interquartile range), and categorical data were described as percentages. Continuous variables were tested using the chi-square tests. A Wilcoxon signed-rank test was used to compare the differences in the D and hemodynamic parameters of the ECA, ICA, VA, and STA before and after surgery. The Mann–Whitney *U*-test was used to analyze the differences between preoperative and postoperative D and hemodynamic parameters in the STA between good and poor collateral development after revascularization surgery. Receiver operator characteristic (ROC) analysis was applied to assess the value of STA ultrasound parameter cutoffs for collateral development. Logistic regression models were used to detect the related factors for surgical collateral development. Statistical analyses were performed using SPSS version 25.0 (SPSS Inc., Chicago, IL, USA). All hypothesis tests were 2-sided, and the significance level was defined at 0.05.

## Results

### The demographic and clinical characteristics of patients

A total of 49 MMD patients were eligible for enrollment, of which 26 patients (18 female patients and eight male patients, aged 37.77 ± 7.42 years old) underwent STA-MCA anastomosis and 23 patients (18 women and five men, aged 33.57 ± 9.83 years old) underwent EDAS surgery. The demographic and clinical characteristics of the patients are listed in Table 1.

**Table 1:** The demographic and clinical characteristics of the MMD patients.

		**STA-MCA anastomosis**	**EDAS**	**P**
Gender, F/M, *n*		18/8	18/5	0.475
Age, years		37.77 ± 7.42	33.57 ± 9.83	0.096
Onset time, months		18.62 ± 23.61	22.39 ± 24.61	0.586
Suzuki Stage, hemispheres	I	0	0	0.182
	II	0	0	
	III	4	4	
	IV	20	13	
	V	2	6	
	VI	0	0	
Hemorrhage/ischemia, *n*		11/15	7/16	0.390
Preoperative MRS score		1.46 ± 0.76	1.96 ± 1.07	0.065
Postoperative MRS score		0.88 ± 0.86	1.26 ± 1.05	0.176
Follow-up period, months		4.21 ± 1.18	4.48 ± 1.15	0.428
Good/poor collateral development, n		19/7	15/8	0.551

MMD, indicates moyamoya disease; STA, superficial temporal artery; MCA, middle cerebral artery; EDAS, encephalo-duro-arterio-synangiosis; F indicates female; M, male; MRS, positive predictive value.

All the items, including age, Suzuki stage, clinical manifestation, preoperative and postoperative MRS scores, onset time, follow-up period, and collateral development, did not show any statistical differences (*P* > 0.05).

### Ultrasonographic changes of the extracranial arteries after STA-MCA anastomosis in MMD patients

**Figure 2:**
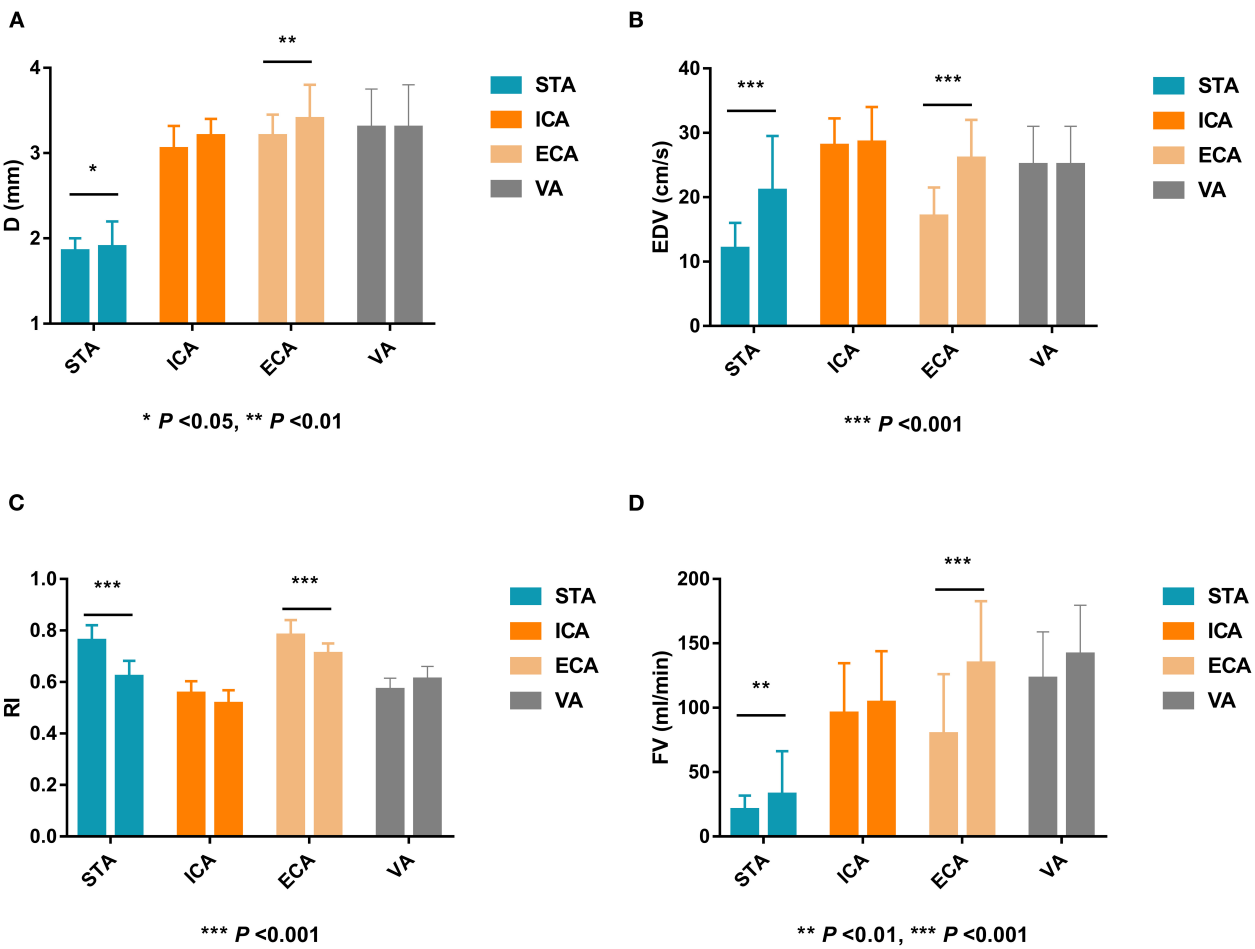
Ultrasonographic changes of extracranial arteries before and after STA-MCA anastomosis in MMD patients. STA, superficial temporal artery; MCA, middle cerebral artery; MMD, indicates moyamoya disease; D, inner diameter; ICA, internal carotid artery; ECA, external carotid artery; VA, vertebral artery; EDV, end-diastolic velocity; RI, resistance index; FV, flow volume.

After STA-MCA anastomosis, the D, EDV, and FV in the STA and ECA were significantly increased compared with those before surgery (*P* < 0.05), and the RI in the STA and ECA were significantly lower than those before surgery (*P* < 0.001). There were no significant differences in ultrasound parameters in the ICA and VA compared with those before STA-MCA anastomosis (*P* > 0.05). Ultrasonographic changes of extracranial arteries in patients after STA-MCA anastomosis are shown in Table 2 and Figure 2.

**Table 2:** Ultrasonographic changes of extracranial arteries before and after STA-MCA anastomosis in MMD patients.

	**Pre-operation**	**Post-operation**	**P**
STA-D (mm)	1.85 (0.42)	1.90 (0.53)	0.029
STA-EDV (cm/s)	12.00 (6.00)	21.00 (13.75)	< 0.001
STA-RI	0.76 (0.11)	0.62 (0.10)	< 0.001
STA-FV (ml/min)	20.50 (17.07)	32.56 (46.42)	0.001
ICA-D (mm)	3.05 (0.65)	3.20 (0.70)	0.909
ICA-EDV (cm/s)	28.00 (13.25)	28.50 (10.00)	0.377
ICA-RI	0.56 (0.20)	0.52 (0.16)	0.807
ICA-FV (ml/min)	95.62 (57.87)	103.95 (92.98)	0.954
ECA-D (mm)	3.20 (0.60)	3.40 (0.65)	0.004
ECA-EDV (cm/s)	17.00 (10.00)	26.00 (13.00)	< 0.001
ECA-RI	0.78 (0.11)	0.71 (0.06)	< 0.001
ECA-FV (ml/min)	79.60 (72.57)	134.43 (71.40)	< 0.001
VA-D (mm)	3.30 (0.55)	3.30 (0.90)	0.418
VA-EDV (cm/s)	25.00 (8.50)	25.00 (0.90)	0.678
VA-RI	0.57 (0.09)	0.55 (0.16)	0.148
VA-FV (ml/min)	122.50 (58.58)	141.47 (91.18)	0.568

STA, superficial temporal artery; MCA, middle cerebral artery; MMD indicates moyamoya disease; D, inner diameter; EDV, end-diastolic velocity; RI, resistance index; FV, flow volume; ICA, internal carotid artery; ECA, external carotid artery; VA, vertebral artery.

### Ultrasonographic changes of extracranial arteries after EDAS surgery in MMD patients

**Figure 3:**
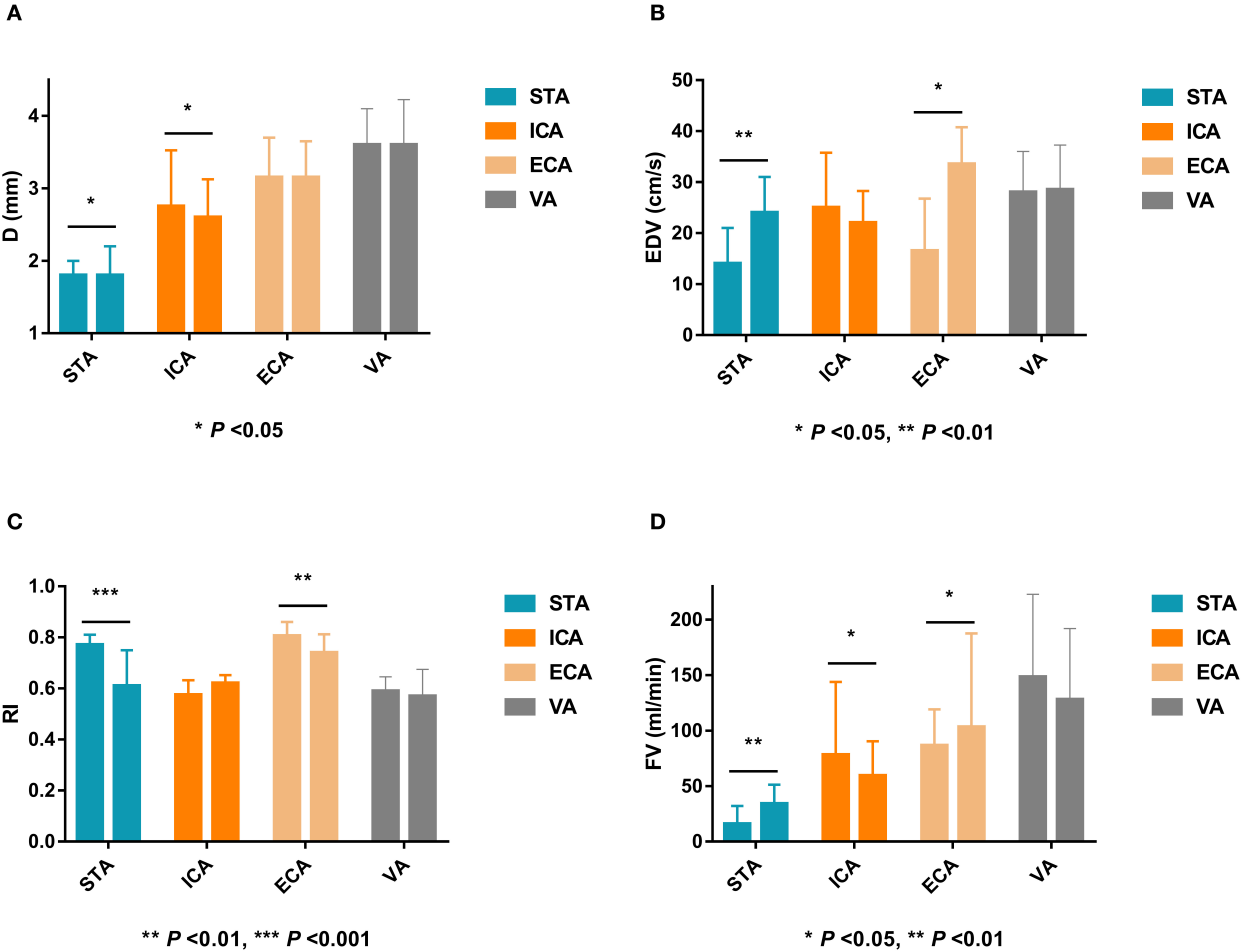
Ultrasonographic changes of extracranial arteries after EDAS in MMD patients. EDAS, encephalo-duro-arterio-synangiosis; MMD, indicates moyamoya disease; D, inner diameter; STA, superficial temporal artery; ICA, internal carotid artery; ECA, external carotid artery; VA, vertebral artery; EDV, end-diastolic velocity; RI, resistance index; FV, flow volume.

After EDAS, the D, EDV, and FV of the STA were significantly higher, and the RI of the STA was significantly lower than that before surgery (*P* < 0.05). The EDV and FV in the ECA after EDAS were significantly higher than those before surgery, and the RI in the ECA after EDAS was significantly lower than that before surgery (*P* < 0.05). The postoperative D and FV in the ICA were significantly lower than before surgery (*P* < 0.05). However, the D, EDV, RI, and FV in the VA after EDAS were not significantly different from those before EDAS (*P* > 0.05). Ultrasonographic changes in extracranial arteries in patients after EDAS are shown in Table 3 and Figure 3.

**Table 3:** Ultrasonographic changes of extracranial arteries after EDAS in MMD patients.

	**Pre-operation**	**Post-operation**	**P**
STA-D (mm)	1.80 (0.60)	1.80 (0.60)	0.038
STA-EDV (cm/s)	14.00 (10.00)	24.00 (15.00)	0.004
STA-RI	0.77 (0.11)	0.61 (0.21)	< 0.001
STA-FV (ml/min)	15.90 (18.74)	34.03 (31.04)	0.003
ICA-D (mm)	2.75 (1.18)	2.60 (0.83)	0.031
ICA-EDV (cm/s)	25.00 (20.00)	22.00 (10.00)	0.407
ICA-RI	0.58 (0.20)	0.62 (0.12)	0.073
ICA-FV (ml/min)	78.17 (115.13)	59.41(53.94)	0.039
ECA-D (mm)	3.20 (1.25)	3.10 (1.05)	0.179
ECA-EDV (cm/s)	17.00 (14.00)	32.00 (22.50)	0.011
ECA-RI	0.80(0.12)	0.73 (0.16)	0.005
ECA-FV (ml/min)	91.56 (63.64)	100.38(106.05)	0.017
VA-D (mm)	3.60 (1.00)	3.60 (1.15)	0.745
VA-EDV (cm/s)	28.00 (16.50)	29.00 (17.50)	0.177
VA-RI	0.59 (0.15)	0.57 (0.20)	0.506
VA-FV (ml/min)	148.30 (120.16)	135.79 (114.36)	0.140

EDAS, encephalo-duro-arterio-synangiosis; MMD, indicates moyamoya disease; STA, indicates superficial temporal artery; D, inner diameter; EDV, end-diastolic velocity; RI, resistance index; FV, flow volume; ICA, internal carotid artery; ECA, external carotid artery; VA, vertebral artery.

### Differences in STA ultrasound parameters between good and poor collateral development groups after STA-MCA anastomosis

**Figure 4:**
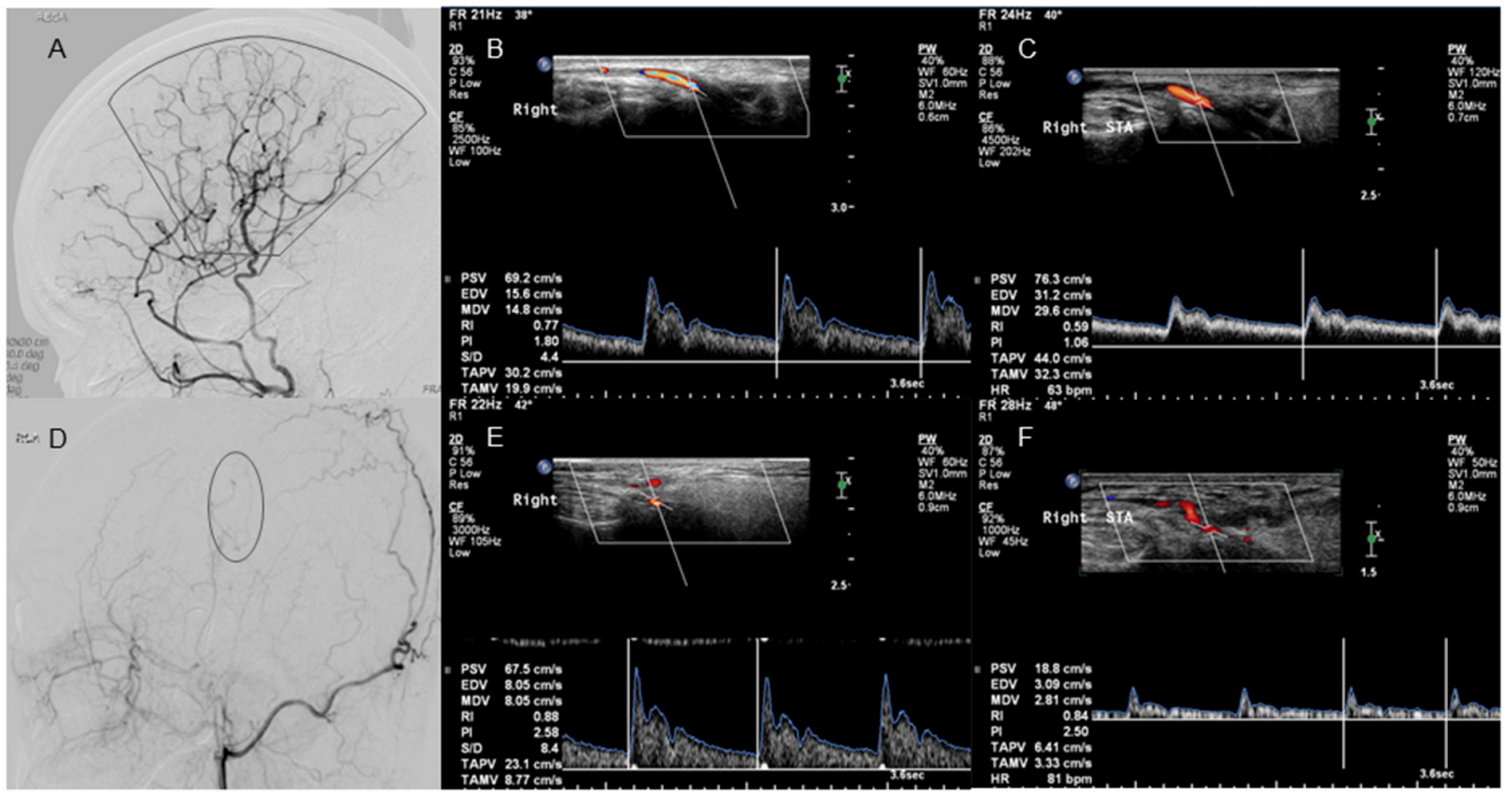
Lateral projection of ECA angiogram and STA ultrasound images in patients with good and poor collateral development after STA-MCA anastomosis. No. 1 A 37-year-old case with moyamoya disease who presented with decreased visual acuity and right-sided motor weakness. **(A)** The lateral projection of the ECA angiogram shows that the perfusion range (sector) of STA collateral vessels exceeds 2/3 of the MCA blood supply area in patients with good collateral development after STA-MCA anastomosis. **(B)** CDU shows the high RI in the STA before STA-MCA anastomosis. **(C)** CDU shows the low RI with increased EDV in the STA after STA-MCA anastomosis in patients with good collateral development. No. 2 A 40-year-old case with moyamoya disease who presented with motor weakness on the right side. **(D)** The lateral projection of the ECA angiogram shows that the perfusion range (oval) of STA collateral vessels is < 2/3 of the MCA blood supply area in patients with poor collateral development after STA-MCA anastomosis. **(E)** CDU shows that the STA has a high RI before STA-MCA anastomosis. **(F)** CDU shows that the STA still has the high RI with low EDV in patients with good collateral development after STA-MCA anastomosis. ECA, external carotid artery; STA, superficial temporal artery; MCA, middle cerebral artery; CDU, color Doppler sonography; RI, resistance index; EDV, end-diastolic velocity.

**Figure 5:**
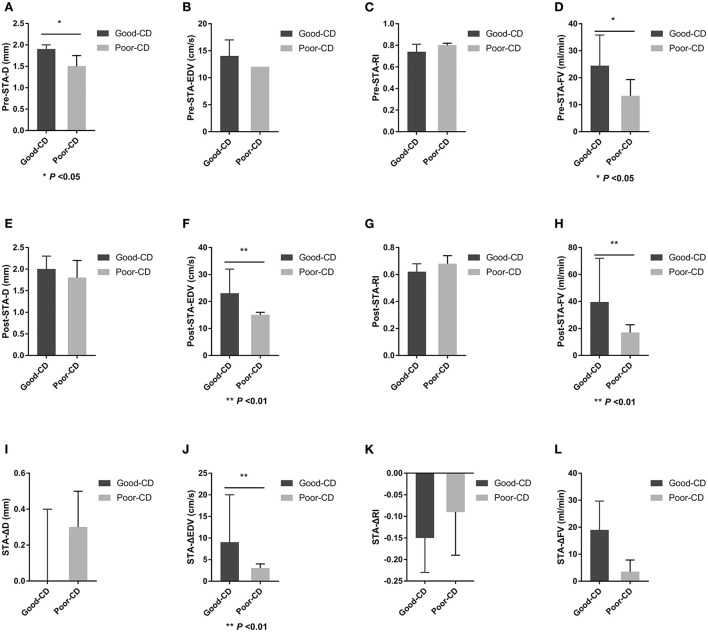
Differences in STA ultrasound parameters between good and poor collateral development groups after STA-MCA anastomosis. STA, superficial temporal artery; MCA, middle cerebral artery; D, inner diameter; CD, collateral development; EDV, end-diastolic velocity; RI, resistance index; FV, flow volume; Δ, the difference between postoperative and preoperative parameters.

There were 19 patients in the good collateral development group and seven patients in the poor collateral development group after STA-MCA anastomosis. The preoperative D and FV of the STA in the good collateral development group were higher than those in the poor collateral development group (*P* < 0.05). The postoperative EDV and FV of the STA in the good collateral development group were higher than those in the poor collateral development group (*P* < 0.01). The EDV difference before and after surgery in the good collateral development group was higher than that in the poor collateral development group (*P* < 0.01). The rest of the parameters had no significant differences (*P* > 0.05; Figure 4). Differences in STA ultrasound parameters between the two subgroups are shown in Table 4 and Figure 5.

**Table 4:** Differences in STA ultrasound parameters between good and poor collateral development groups after STA-MCA anastomosis.

	**Good collateral development**	**Poor collateral development**	**P**
	**(*n* = 19)**	**(*n* = 7)**	
Preoperative STA-D (mm)	1.90 (0.20)	1.50 (0.40)	0.013
Preoperative STA-EDV (cm/s)	14.00 (7.00)	12.00 (2.00)	0.135
Preoperative STA-RI	0.74 (0.11)	0.80 (0.07)	0.188
Preoperative STA-FV (ml/min)	24.42 (17.09)	13.26 (8.98)	0.048
Postoperative STA-D (mm)	2.00 (0.60)	1.80 (0.60)	0.188
Postoperative STA-EDV (cm/s)	23.00 (13.00)	15.00 (3.00)	0.002
Postoperative STA-RI	0.62 (0.22)	0.68 (0.13)	0.073
Postoperative STA-FV (ml/min)	39.47 (47.95)	16.88 (8.09)	0.003
STA-ΔD (mm)	0.00 (0.60)	0.30 (0.30)	0.169
STA-ΔEDV (cm/s)	9.00 (12.00)	3.00 (3.00)	0.007
STA-ΔRI	−0.15 (0.15)	−0.09 (0.12)	0.209
STA-ΔFV (ml/min)	18.95 (18.08)	3.47 (9.74)	0.063

STA, superficial temporal artery; MCA, middle cerebral artery; D, inner diameter; EDV, end-diastolic velocity; RI, resistance index; FV, flow volume; Δ, the difference between postoperative and preoperative parameters.

### Differences in STA ultrasound parameters between good and poor collateral development groups after EDAS

**Figure 6:**
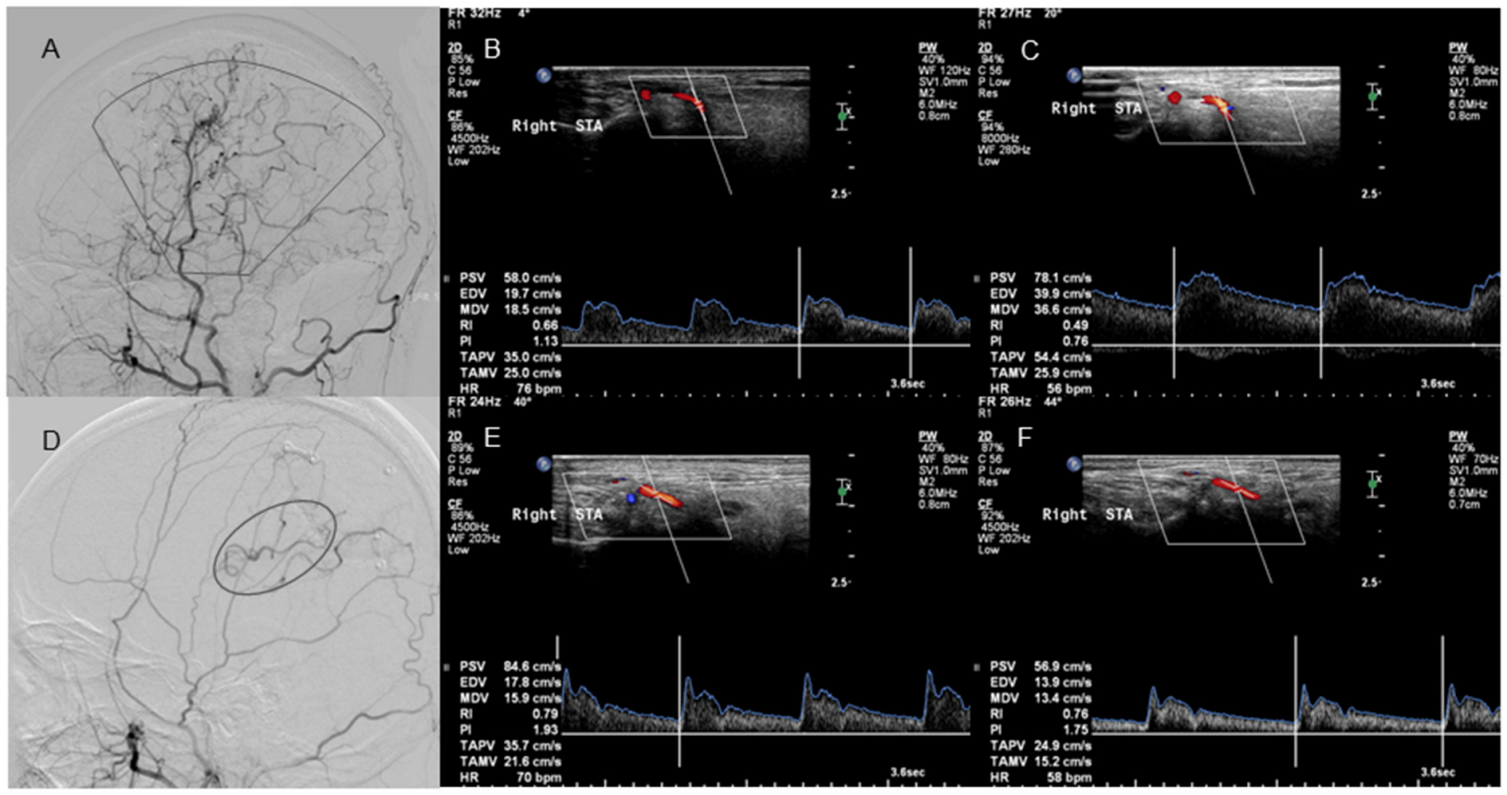
Lateral projection of ECA angiogram and STA ultrasound images in patients with good and poor collateral development after EDAS. No. 1 A 25-year-old case with moyamoya disease who presented with motor weakness on the right side. **(A)** The lateral projection of the ECA angiogram shows that the perfusion range (sector) of STA collateral vessels exceeds 2/3 of the MCA blood supply area after EDAS in patients with good collateral development. **(B)** CDU shows the high RI in the STA before EDAS. **(C)** CDU shows the low RI with increased EDV in the STA after EDAS in patients with good collateral development. No. 2 A 22-year-old case with moyamoya disease who had a language disability. **(D)** The lateral projection of the ECA angiogram shows that the perfusion range (oval) of STA collateral vessels is < 2/3 of the MCA blood supply area after EDAS in patients with poor collateral development. **(E)** CDU shows that the STA has a high RI before EDAS. **(F)** CDU shows that the STA still has the high RI with low EDV after EDAS in patients with good collateral development. ECA, external carotid artery; STA, superficial temporal artery; EDAS, encephalo-duro-arterio-synangiosis; MCA, middle cerebral artery; CDU, color Doppler sonography; RI, resistance index; EDV, end-diastolic velocity.

**Figure 7:**
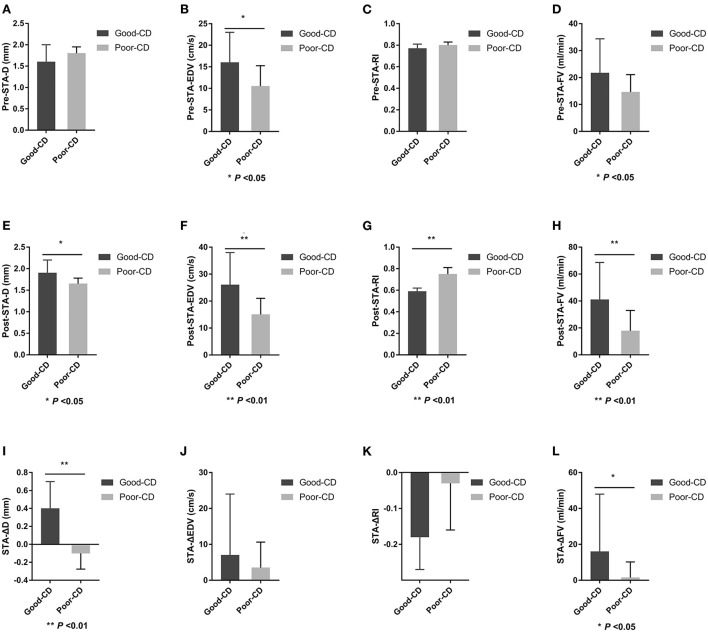
Differences in STA ultrasound parameters between good and poor collateral development groups after EDAS. STA, superficial temporal artery; EDAS, encephalo-duro-arterio-synangiosis; D, inner diameter; CD, collateral development; EDV, end-diastolic velocity; RI, resistance index; FV, flow volume; Δ, the difference between postoperative and preoperative parameters.

There were 15 patients in the good collateral development group and eight patients in the poor collateral development group after EDAS. The preoperative EDV of the STA in the good collateral development group was higher than those in the poor collateral development group (*P* < 0.05). The postoperative D, EDV, and FV of the STA in the good collateral development group were higher than those in the poor collateral development group. The postoperative RI was lower than that in the poor collateral development group (*P* < 0.05). The D difference and FV difference before and after surgery in the good collateral development group were higher than those in the poor collateral development group (*P* < 0.05). The rest of the parameters had no significant differences (*P* > 0.05; Figure 6). Differences in STA ultrasound parameters between the two groups are shown in Table 5 and Figure 7.

**Table 5:** Differences in STA ultrasound parameters between good and poor collateral development groups after EDAS.

	**Good collateral development**	**Poor collateral development**	**P**
	**(*n* = 15)**	**(*n* = 8)**	
Preoperative STA-D (mm)	1.60 (0.80)	1.80 (0.653)	0.875
Preoperative STA-EDV (cm/s)	16.00 (10.00)	10.50 (8.50)	0.040
Preoperative STA-RI	0.77 (0.15)	0.80 (0.11)	0.238
Preoperative STA-FV (ml/min)	21.70 (20.91)	14.62 (7.88)	0.428
Postoperative STA-D (mm)	1.90 (0.40)	1.65 (0.58)	0.019
Postoperative STA-EDV (cm/s)	26.00 (17.00)	15.00 (10.00)	0.008
Postoperative STA-RI	0.59 (0.09)	0.75 (0.15)	0.008
Postoperative STA-FV (ml/min)	41.03 (40.91)	17.81 (19.98)	0.005
STA-ΔD (mm)	0.40 (0.50)	−0.10 (0.28)	0.002
STA-ΔEDV (cm/s)	7.00 (24.00)	3.50 (16.13)	0.238
STA-ΔRI	−0.18 (0.22)	−0.03 (0.17)	0.065
STA-ΔFV (ml/min)	16.04 (37.14)	1.53 (12.24)	0.016

STA, superficial temporal artery; EDAS, encephalo-duro-arterio-synangiosis; D, inner diameter; EDV, end-diastolic velocity; RI, resistance index; FV, flow volume; Δ, the difference between postoperative and preoperative parameters.

### Cutoff values and AUC (area under the ROC curve) for predicting and evaluating the collateral development after STA-MCA anastomosis

**Figure 8:**
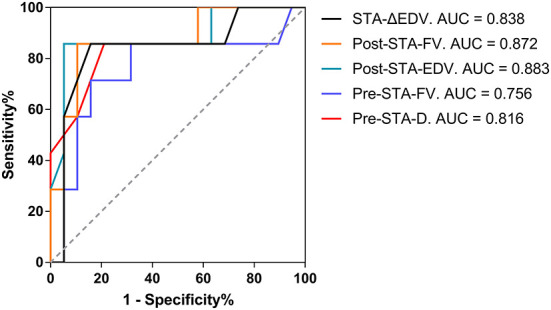
The AUC for the collateral development after STA-MCA anastomosis. AUC, an area under the Receiver Operating Characteristic curve; STA, superficial temporal artery; MCA, middle cerebral artery; Δ, the difference between postoperative and preoperative parameters; EDV, end-diastolic velocity; Post, postoperative; FV, flow volume; Pre, preoperative; D, inner diameter.

Preoperative D > 1.75 mm in the STA as the cutoff value for predicting the collateral development had the highest AUC of 0.816 with 78.95% sensitivity, 85.71% specificity, 93.75% PPV, and 60.00% NPV, and 80.77% overall accuracy. Besides, the highest AUC with FV in the STA was 0.756, as the clinical predicting index for collateral development. After STA-MCA anastomosis, postoperative EDV > 16.50 cm/s in the STA as the cutoff value for evaluating the collateral development had the highest AUC of 0.883 with 94.74% sensitivity, 85.71% specificity, 94.74% PPV, and 85.71% NPV, and 92.31% overall accuracy. Meanwhile, as the clinical evaluation index for collateral development, the highest AUC with EDV and FV in the STA was 0.872 and 0.838 (Table 6 and Figure 8).

**Table 6:** The cutoff values and AUC for the collateral development after STA-MCA anastomosis.

	**Cutoff**	**Overall accuracy (%)**	**Sensitivity (%)**	**Specificity (%)**	**PPV (%)**	**NPV (%)**	**AUC (95% CI)**	**P**
Preoperative STA-D (mm)	1.75	80.77	78.95	85.71	93.75	60.00	0.816 (0.578–1.000)	0.015
Preoperative STA-FV (ml/min)	16.43	80.77	84.21	71.43	88.89	62.50	0.756 (0.515–0.997)	0.049
Postoperative STA-EDV (cm/s)	16.50	92.31	94.74	85.71	94.74	85.71	0.883 (0.711–1.000)	0.003
Postoperative STA-FV (ml/min)	22.97	88.46	89.47	85.71	94.44	75.00	0.872 (0.708–1.000)	0.004
STA-ΔEDV (cm/s)	5.00	84.62	84.21	85.71	94.12	66.67	0.838 (0.642–1.000)	0.009

STA, superficial temporal artery; MCA, middle cerebral artery; PPV, indicates positive predictive value; NPV, negative predictive value; AUC, area under the Receiver Operating Characteristic curve; CI, confidence interval; D, inner diameter; FV, flow volume; EDV, end-diastolic velocity; Δ, the difference between postoperative and preoperative parameters.

### Cutoff values and AUC for predicting and evaluating the collateral development after EDAS

**Figure 9:**
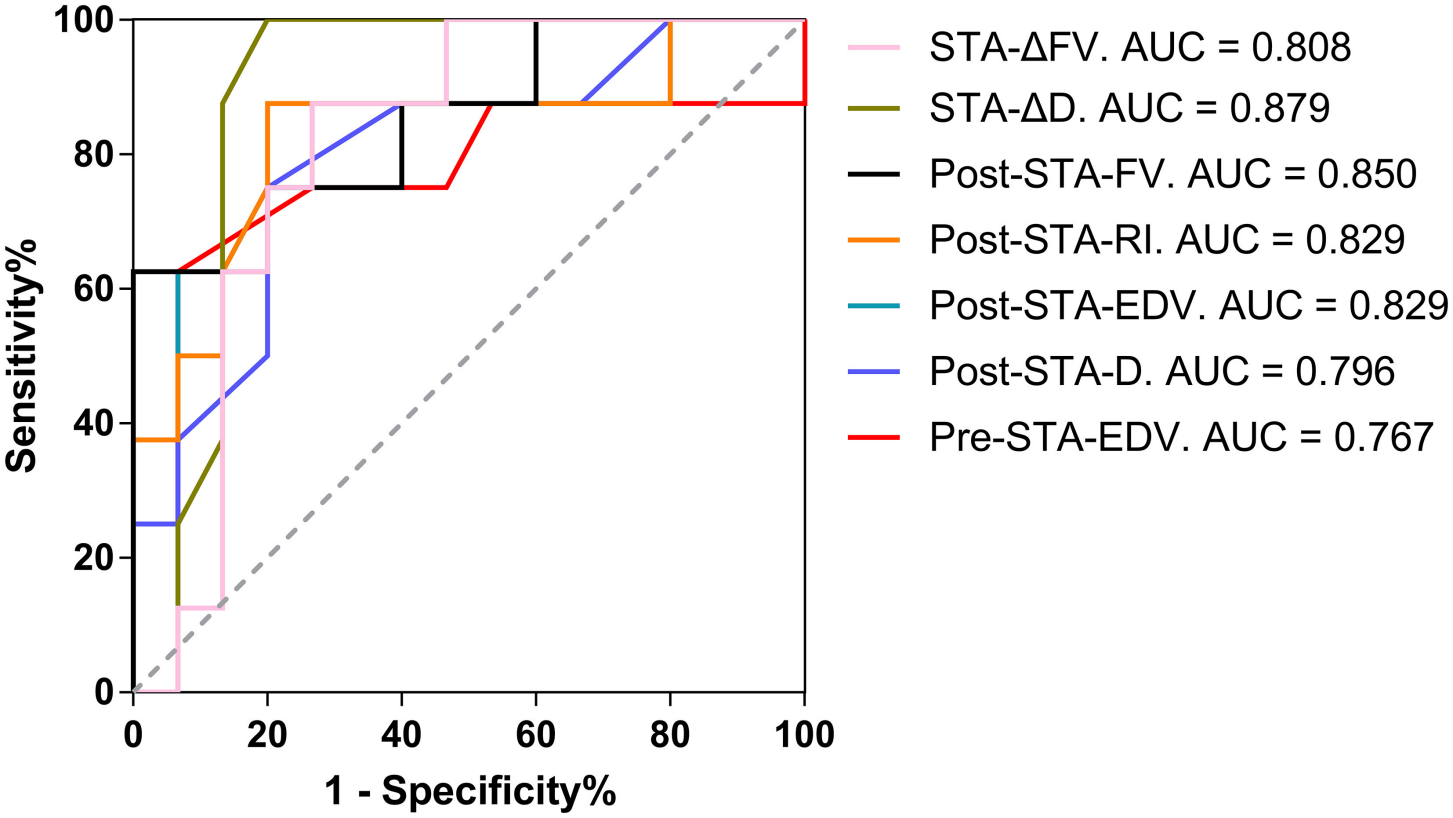
The AUC for the collateral development after EDAS. AUC, area under the Receiver Operating Characteristic curve; EDAS, encephalo-duro-arterio-synangiosis; STA, superficial temporal artery; Δ, the difference between postoperative and preoperative parameters; FV, flow volume; D, inner diameter; Post, postoperative; RI, resistance index; EDV, end-diastolic velocity; Pre, preoperative.

Preoperative EDV > 12.00 cm/s in the STA as the cutoff value for predicting collateral development had the highest AUC of 0.767 with 93.33% sensitivity, 62.50% specificity, 82.35% PPV, and 83.88% NPV, and 82.61% overall accuracy. After EDAS, ΔD > 0.15 mm in the STA as the cutoff value for the collateral development had the highest AUC of 0.879 with 80.00% sensitivity, 100.00% specificity, 100.00% PPV, 72.73% NPV, and 86.96% overall accuracy, which were higher than the other ultrasound parameters in the STA as the clinical indexes (Table 7 and Figure 9).

**Table 7:** The cutoff values and AUC for the collateral development after EDAS.

	**Cutoff**	**Overall accuracy (%)**	**Sensitivity (%)**	**Specificity (%)**	**PPV (%)**	**NPV (%)**	**AUC (95% CI)**	***P***
Preoperative STA-EDV (cm/s)	12.00	82.61	93.33	62.50	82.35	83.88	0.767 (0.523, 1.000)	0.039
Postoperative STA-D (mm)	1.75	78.26	80.00	75.00	85.71	66.67	0.796 (0.598, 0.993)	0.022
Postoperative STA-EDV (cm/s)	23.00	87.26	73.33	87.50	91.67	63.64	0.829 (0.632, 1.000)	0.011
Postoperative STA-RI	0.64	82.61	80.00	87.50	92.31	70.00	0.829 (0.632, 1.000)	0.011
Postoperative STA-FV (ml/min)	26.87	78.26	80.00	75.00	85.71	66.67	0.850 (0.676, 1.000)	0.003
STA-ΔD (mm)	0.15	86.96	80.00	100.00	100.00	72.73	0.879 (0.724, 1.000)	0.017
STA-ΔFV (ml/min)	11.46	78.26	73.33	87.50	91.67	63.64	0.808 (0.627, 0.990)	0.017

EDAS, encephalo-duro-arterio-synangiosis; PPV, positive predictive value; NPV, negative predictive value; AUC, area under the Receiver Operating Characteristic curve; CI, confidence interval; STA, superficial temporal artery; EDV, end-diastolic velocity; D, inner diameter; RI, resistance index; FV, flow volume; Δ, the difference between postoperative and postoperative parameter.

### The association of preoperative STA parameters with collateral development after revascularization surgery

Preoperative STA parameters D, EDV, RI, and FV as the independent variables were put into the binary logistic regression equation with collateral development as the dependent variable for univariate and multivariate analysis. Logistic-related factor analysis showed that the RI of preoperative STA was negatively correlated with collateral development after adjusting for patient age (*P* < 0.05). After adjusting for patient age, Suzuki stage, and surgical method, the RI of preoperative STA was negatively and EDV was positively correlated with collateral development (*P* < 0.05; Table 8). Besides, the RI of STA before surgery was negatively correlated with the collateral development after revascularization in the multivariate analysis (*P* < 0.05).

**Table 8:** The association of preoperative STA parameters with collateral development after revascularization surgery.

**Parameter**	**Univariate analysis** ^**#**^			Corrected mode 1*			**Corrected mode 2^†^**			**Multivariate analysis^‡^**		
	**OR**	**95% CI**	**P**	**OR**	**95% CI**	**P**	**OR**	**95% CI**	**P**	**OR**	**95% CI**	**P**
STA-D	3.60	0.603–21.450	0.160	4.30	0.66–27.91	0.126	3.85	0.57–25.97	0.166	-	-	-
STA-EDV	1.15	0.99–1.34	0.067	1.16	0.99–1.36	0.061	1.22	1.03–1.44	0.023	-	-	-
STA-RI	2.50E-5	4.66E-10–1.33	0.057	3.00E-6	2.10E-11–0.50	0.038	8.62E-7	3.56E-12–0.21	0.027	4.00E-6	4.21E-11–0.428	0.035
STA-FV	1.05	0.99–1.11	0.112	1.06	0.992–1.12	0.093	1.06	0.994–1.13	0.076	-	-	-

STA, superficial temporal artery; OR, odds ratio; CI, confidence interval; D, inner diameter; EDV, end-diastolic velocity; RI, resistance index; FV, flow volume.

^**#**^Univariate analysis: the univariate binary logistic regression method was used.

^*^Corrected mode 1: adjusting for patient age.

^†^Corrected mode 2: adjusting for patient age, Suzuki stage, and surgical method.

^‡^Multivariate analysis: the multivariate binary logistic regression method based on likelihood ratio was used.

## Discussion

Our study showed that the D increased, EDV increased, RI decreased, and FV increased in the STA and ECA after STA-MCA anastomosis and EDAS. Yeh and Jin et al. found that the blood flow parameters of STA and ECA in patients with MMD after successful revascularization had obvious changes, such as increased blood flow velocity, decreased resistance, and pulsatility index. The changes began in the 2nd week after EDAS (19, 23, 24). However, the changes could appear immediately after STA-MCA anastomosis (25, 26). In our study, the changes in various ultrasound indexes of ECA and STA after surgery were consistent with previous studies, indicating that STA has established collateral circulation to the brain after STA-MCA anastomosis and EDAS.

On this basis, the ultrasonographic changes in the VA and ICA after surgery were also studied in our study. The results showed that VA blood flow did not change obviously after surgery. Interestingly, ICA hemodynamic changes were different after STA-MCA anastomosis and after EDAS. We found the ICA parameters did not change significantly after STA-MCA anastomosis, while ICA inner diameter decreased and FV decreased after EDAS. It may be related to the strong collateral angiogenesis and compensation ability of STA from the brain surface to the inside of EDAS patients, resulting in decreased demand for the blood supply from the ICA (18). On the contrary, the intracranial blood supply *via* STA anastomosis with MCA branches may not be sufficient to reduce the demand for blood supply from the ICA. Besides, EDAS might be responsible for the disease progression of anterior circulation stenosis. Thus, we speculated that STA-MCA anastomosis could delay disease progression. However, further research is needed.

During anterior circulation ischemia in MMD patients, the vertebral-basilar-posterior cerebral artery is a vital blood supply source for the brain. The posterior cerebral artery supplies blood for the anterior circulation through the leptomeningeal cortical branches to relieve its insufficiency (18, 27, 28). In this study, VA ultrasound parameters did not change after surgery, indicating that the blood supply increase in the anterior circulation might not be enough to reduce the need for posterior circulation compensation after STA-MCA anastomosis or EDAS in the short term.

This study analyzed the differences in ultrasound parameters between good and bad collateral development patients. The D, EDV, and FV of the STA in the good collateral development group after EDAS increased significantly compared with those in the poor, and the RI decreased considerably. The results suggested that the STA developed more neovascularization in the brain in the good collateral development group after EDAS than in the poor. According to our results, FV increases in the STA by increasing the D and velocity supply to the neovascularization. When STA supplies more blood to the brain, the patient's clinical symptoms could improve, and the occurrence of adverse events would decrease. Yeh et al. (19) observed the changes in STA ultrasound parameters after surgery in 21 patients with unilateral or bilateral indirect vascular reconstruction surgery (including EDAS, etc.). They proposed that an increase in EDV of 13.5 cm/s and a decrease in RI of 0.19 have relatively high sensitivity and specificity for evaluating the collateral development of indirect cerebrovascular reconstruction. Unlike previous studies, our results showed that the inner diameter had the most obvious change. Using an increase of D of 0.15 mm after surgery in the STA as the critical index to evaluate the postoperative collateral development of EDAS, the sensitivity and specificity were higher than those of EDV, FV, and other indicators. The possible reasons are that the surgical method and operation times differed between the two studies. First, we selected patients with MMD who underwent revascularization for the first time. Besides, the indirect revascularization surgery was only limited to EDAS. Craniotomy, encephalo-myo synangiosis (EMS), and other indirect revascularization procedures and occipital artery-MCA, STA-ACA, and other direct revascularization procedures were not included in our study.

In contrast to the patients with EDAS, no significant difference in the D of the STA was found after STA-MCA anastomosis, while the EDV and FV in the good collateral development group were significantly higher than those in the poor collateral development group, so we believed the increase in the FV of the STA may depend on the EDV, not the D. It is possible that, in addition to gradually forming new blood vessels around the STA like EDAS, the blood from STA could also use some remaining MCA branches to establish the intracranial blood supply channels immediately after STA-MCA anastomosis (26). Although some symptoms of patients can be relieved in time after STA-MCA anastomosis, there is also a risk of intracranial hyperperfusion. That's why young patients with great potential to develop collaterals tend to perform EDAS in case of intracranial hyperperfusion (18). Among the various ultrasound indicators, the change of EDV after STA-MCA anastomosis was the most obvious, and the sensitivity and specificity of EDV > 16.5 cm/s as the cutoff value to evaluate the collateral development was higher than that of FV and others. Taken together, monitoring the EDV in the STA after STA-MCA anastomosis and D in the STA after EDAS could be useful for evaluating surgical collateral development. When the patients have a lower postoperative EDV in the STA after STA-MCA anastomosis or a smaller D in the STA after EDAS, more attention should be paid to the possibility of poor collateral development.

This study also revealed that the preoperative EDV and RI in the STA had a positive and negative correlation with the collateral development, respectively, when adjusted for age, Suzuki stage, and surgical method. This may be related to the vascular elasticity of the STA itself or the ability of the STA to regenerate collateral circulation (18). In the multivariant analysis, low preoperative RI in the STA was the independent factor of collateral development. Perhaps, high EDV and low RI before surgery indicate that the STA has good vascular elasticity, a strong ability to spontaneously form collaterals or some new collaterals that have formed in the brain, so that it could supply more collateral vessels to the brain after surgery, and vice versa (Figures 4, 6). Our study also showed that the preoperative D and FV of the STA in the good collateral development group were obviously higher than those in the poor after STA-MCA anastomosis. In the ROC curve analysis, the results also indicated that both the preoperative D and FV of the STA had an accuracy of 80.77% as the predictive indicator of collateral development after STA-MCA anastomosis. Similarly, we also found that the difference in preoperative EDV in the STA in the good collateral development group was higher than that in the poor, which had an accuracy of 82.61% as the predictive indicator of collateral development after EDAS. These results might suggest that it is feasible to assist clinicians in using preoperative ultrasound parameters to predict the postoperative curative effect on patients.

Our study also has some limitations. First, selection bias could affect the sample because of the inclusion conditions and the loss of follow-up in some patients. However, we attempted to minimize selection bias by collecting consecutive patients. Ultrasound also couldn't quantitatively assess the amount of postoperative neovascularization. In addition, no longer-term follow-up for the postoperative patients was performed. Another disadvantage of this study is that the clinician determined the patient's surgical method in advance based on the patient's clinical and hemodynamic data, so their comparisons with postoperative collateral development for each surgery could not be studied. Finally, this study did not combine cerebral perfusion examination with prognostic analysis, which needs further improvement in subsequent studies.

In conclusion, EDAS and STA-MCA anastomosis have different effects on the anterior circulation, but they did not have a noticeable effect on the posterior circulation. CDU could be used as a non-invasive method to monitor STA parameters before and after surgery for preoperative prediction and postoperative evaluation of surgical collateral development in MMD patients. Future prospective studies with large sample sizes and long-term follow-up are needed to confirm our findings.

## Data availability statement

The data that support the findings of this study are available from the corresponding author upon reasonable request.

## Ethics statement

The studies involving human participants were reviewed and approved by the Research Ethics Board of the Beijing Tiantan Hospital, Capital Medical University. Written informed consent to participate in this study was provided by the patients/participants.

## Author contributions

J-ZW designed and wrote the manuscript. JM and SZ drafted the pictures. DZ provided radiological resources. XZ and XW revised it critically for intellectual content. All authors reviewed and approved the final manuscript.

## Funding

This work was sponsored by Tianjin Health Research Project, Tianjin, China (Grant number: TJWJ2022QN012).

## Conflict of interest

The authors declare that the research was conducted in the absence of any commercial or financial relationships that could be construed as a potential conflict of interest.

## Publisher's note

All claims expressed in this article are solely those of the authors and do not necessarily represent those of their affiliated organizations, or those of the publisher, the editors and the reviewers. Any product that may be evaluated in this article, or claim that may be made by its manufacturer, is not guaranteed or endorsed by the publisher.
